# Higher Prevalence of Sexual Transmitted Diseases and Correlates of Genital Warts among Heterosexual Males Attending Sexually Transmitted Infection Clinics (MSCs) in Jiangmen, China: Implication for the Up-Taking of STD Related Service

**DOI:** 10.1371/journal.pone.0121814

**Published:** 2015-03-26

**Authors:** Shujie Huang, Weiming Tang, Zhengjun Zhu, Hekun Lu, Xueling Tan, Baoyuan Zhang, John Best, Ligang Yang, Heping Zheng, Ning Jiang, Yueping Yin, Bin Yang, Xiangsheng Chen

**Affiliations:** 1 Guangdong Provincial Center for Skin Diseases and STI Control, No. 2 Lujing Road, Guangzhou, 510095, China; 2 University of North Carolina Project-China, No. 2 Lujing Road, Guangzhou, 510095, China; 3 Jiangmen Dermatology Hospital, No. 62 Yuejin Road, Jiangmen, 529000, China; 4 Department of Medicine, Medical School, University of California San Francisco, San Francisco, CA, 94143, United States of America; 5 National Center for STD Control, China CDC. No. 12, Jiangwangmiao, Nanjing, Jiangsu, 210000, China; China Medical University, CHINA

## Abstract

**Background:**

Increasing burden of STDs is one of China’s major public health concerns. However, only a limited number of studies have ever investigated the prevalence of these STDs, particular for genital warts and its correlates among heterosexual males attending STD clinics in China. In order to fill this gap, we conducted a cross-sectional study among MSCs in Jiangmen, China, between the years of 2009 and 2010.

**Method:**

The eligible participants were recruited from several STD-clinics in public hospitals. We collected demographic information and behaviors of the participants. After HIV and syphilis testing, we further checked whether the participants had genital warts and genital herpes. In addition, urine samples were collected from part of the participants for CT and NG testing.

**Results:**

Of the 533 eligible participants, over three-fifths were aged 35 or below, nearly three quarters had no college degree, over three-fifths were residence of Jiangmen. The prevalence of HIV, syphilis, genital warts, genital herpes, CT and NG were 0.19%, 7.50%, 7.32%, 5.25%, 9.73% and 6.19%, respectively. Living with family members (versus living alone), no STD-related service in past year, experiencing STDs related symptoms in past year, and sex with FSWs in last three months were positively associated with genital warts, with adjusted ORs of 5.54 (95% CI 1.94–15.81), 2.26 (95% CI 1.08–4.74), 1.99 (95% CI 1.00–3.99) and 2.01 (95% CI 1.00–4.04), respectively.

**Conclusion:**

Our study indicates that the prevalence of STDs among MSCs in Jiangmen was high, which may further spread HIV among MSCs. Targeted interventions that focused on STDs related services uptake should be implemented urgently.

## Introduction

It was estimated that in 2008 the total number of new cases of four common STDs (Chlamydia trachomatis (CT), Neisseria gonorrhoeae (NG), syphilis and Trichomonas vaginalis) in adults was about 500 million, an increase of 11% since 2005[1]. Increasing burden of STDs is also one of China’s major public health concerns [2,3]. A number of studies have revealed the prevalence rates of syphilis, HIV, CT, NG, HSV-2 and human papilloma virus (HPV) to be high among Chinese men who have sex with men (MSM) and female sex workers (FSWs)[4–9]. However, only a limited number of studies have ever investigated the prevalence of these STDs among heterosexual males attending STD clinics (MSCs) in China[10].

As a common sexually transmitted disease (STD), Genital warts is caused by non-oncogenic HPV[11]. The prevalence of genital warts is usually high among MSM and FSWs [12,13]. The heavy burden of genital warts has important public health significance, as genital warts are associated with lower health-related quality of life[14], the treatmentis usually expensive[15] and there is a high recurrence rate[16]. In addition, there is only a small number of epidemiological studies that focused on the factors correlated with genital warts worldwide[17].

Given the limited knowledge regarding the prevalence of STDs among MSCs and the lack of studies that explore the factors correlated with genital warts worldwide, a comprehensive epidemiological study is needed to aid in design of appropriate intervention strategies/programs. To fill this knowledge gap, we conducted a cross-sectional study among MSCs in Jiangmen, Guangdong, China, between the years of 2009 and 2010. Jiangmen is a well-developed city located in Zhujiang Delta Area, China. Previous pilot studies conducted in 2008 in Jiangmen suggested that MSCs had high prevalence of STDs, indicating the need for a more comprehensive study.

## Methods

This cross-sectional study was a part of the “China Mega Project”. The current reported study was based on the survey conducted between July 2009 and June 2010 in Jiangmen city of Guangdong province, China.

### Ethical statement

The Ethics Committee of National Center for STD Control of China CDC approved the study process and contents. Signed informed consent was obtained from each of the participants prior to the interviews and disease diagnosis. Each participant was free to decline or withdraw from the study at any time.

### Recruitment

The participants were recruited from seven STD-clinics in public hospitals throughout Jiangmen using the convenience sampling method. Participants who were born male, 18 years old or older, attended the STD-clinics for health care services during the recruitment period, had female partners in last three months, were not engaged in sex with men in the last three months and provided informed consent were included in the study. Participants who were unable to participate actively due to medical reasons or those who were engaged in sex with other men in the past three months were excluded from the survey.

### Structured Interview

After the assessment of eligibility and collection of informed consents, venous blood samples were collected from each participant for free HIV and syphilis testing. Next, interviewer administered questionnaire-based interviews were conducted to collect demographic information such as age, education level (senior high school or below/ college or higher), marital status (never married/ ever married), residency status (official residence of Jiangmen/ migrants). Participants were also asked about recent sexual behaviors, including condom during last vaginal intercourse with FSWs, casual partners, and regular partners. STD related symptoms were measured by asking whether participants experienced any of the listed symptoms (burning pain when urinating, genital discharge, ulcer/sores on penis/anus and genital growth) in the last year. STD related service was defined by asking whether the participants received any of the following service in the last year: STD testing and treatment, education on HIV/STDs, condom distribution or partner notification. STD related service was generally provided health providers. In our study, living with family members was defined as living with parents or other family members except female partners. Living with others means living people other than family members and female partners, for example, including friends.

### Laboratory testing

The participants were tested for HIV, syphilis, CT and NG. The participants were first screened for HIV-1 or HIV-2 by using rapid test (using ELISA kit, Livzon Diagnostic Inc., Zhuhai). Blood samples tested positive in the rapid test were confirmed with HIV Blot 2.2(MP Biomedicals Asia Pacfic Pte. Ltd, Singapore). Samples positive for syphilis screening (using ELISA kit, Wantai Biological Pharmacy Enterprise Co., Ltd, Beijing) were confirmed by the Toluidine Red Untreated Serum Test (TRUST, Wantai Biological Pharmacy Enterprise Co.,Ltd). In our study, participants diagnosed positive for the Western Blot test for HIV antibody were defined as HIV-positive. In addition, participants who were positive for both Toluidine Red Unheated Serum Test and ELISA were defined as currently syphilis positive [18,19]. STD-clinic physicians appropriately treated all participants diagnosed (through physical examinations and blood testing) with any STD. Participants who tested positive for HIV were referred to national HIV care and treatment program for further follow up and required treatment, according to the protocol of “China Mega Project”.

Urine samples of 226 participants were also collected for CT and NG testing at the National STD Reference Laboratory at Nanjing by using Polymerase Chain Reaction (PCR: Roche Amplicor assay, Roche Diagnostic Systems, Indianapolis, IN).

### Physical Examination and Testing Result Disclosure

After the survey, every participant was further assessed for genital warts and genital herpes by clinical diagnosis method. In this step, the physicians performed physical examinations to each participant, to check whether they had genital warts and genital herpes. Following the national guidelines, the HIV and syphilis testing results were then declared to the participants in a private room where the STD-clinic physicians conducted physical examination and provided counseling to each of the participants. The testing results of CT/NG results were declared to the participants one week later, while potential treatment was given to the participants who had CT or NG-related symptoms right after the survey.

### Data Analysis

Data were double entered into Epidata 3.02. Multiple logic checks were used to check the data consistency with Microsoft Excel and STATA 10.0. SAS statistical analysis software version 9.3 [20] was used to describe the demographic and sexual behaviors, to estimate the prevalence of syphilis, HIV, genital herpes, genital warts, CT and NG. Simple logistic regression was first used to identify the factors correlating with genital warts and calculate crude odds ratio (cOR) and 95% confidence intervals (95%CI). Multiple logistic regressions were performed with adjustment for age (continuous), residence (local/migrants), marital status (ever married/never married) and education (senior high school or below/college or above).

## Results

Between the year of 2009 and 2010, a total of 533 eligible MSCs were recruited and completed the survey in Jiangmen, China.

### Socio-demographic and sexual behaviors

Among participants, over three-fifths (61.16%) were aged 35 or below, nearly three quarters (72.61%) had no college degree, over three-fifths (62.03%) were residence of Jiangmen. About one third were never married (32.08%) while over three-fifths (61.73%) of the eligible participants currently lived with female partners.

About half (48.49%) of the participants reported experiencing listed STDs symptoms in the last year, while only about 45% of them ever received any STDs related service in the last year.

In our study, more than half (54.73%) of the participants reported engaging in sex with FSWs in the last three months. In addition, more than two fifths (40.72%) of the participants reported had sex with casual female partners in the last three months. When engaged in sex with regular female partners, 51.97% participants reported that unprotected sex during last intercourse. This number is even higher when engaged in sex with FSWs and casual female partners, with rates of unprotected sex of 73.73% and 77.67%, respectively (Table 1).

**Table 1:** Socio-demographic and STDs risky behaviors of among heterosexual males attending sexually transmitted infection clinics in Jiangmen, China, 2009 (N = 533).

**Variables**	**Frequency**	**Percent**
**Age**		
*25 and below*	121	22.70
*26–35*	205	38.46
*36–45*	120	22.51
*Above 45*	87	16.32
**Education**		
*Junior High school or below*	387	72.61
*College or above*	146	27.39
**Residence**		
*Local*	330	62.03
*Migrants*	202	37.97
**Marital status**		
*Never married*	171	32.08
*Ever married*	362	67.92
**Living status**		
*Living alone*	138	25.89
*Live with female partners*	329	61.73
*Live with others*	21	3.94
*Live with family members*	45	8.44
**Experienced listed STDs related symptoms in the last year**		
*Yes*	257	48.49
*No*	273	51.51
**Received STDs related service in the past year**		
*No*	291	54.6
*Yes*	242	45.4
**Used condom with regular female partners during the last intercourse**		
*No*	277	51.97
*Yes*	256	48.03
**Used condom with female sex workers during the last intercourse**		
*No*	393	73.73
*Yes*	140	26.27
**Used condom with casual female partners during the last intercourse**		
*No*	414	77.67
*Yes*	119	22.33
**Had sex with female sex workers in the last 3 months**		
*No*	239	45.27
*Yes*	289	54.73
**Had sex with casual female partners in the last 3 months**		
*No*	313	59.28
*Yes*	215	40.72

### STDs Prevalence

**Fig 1:**
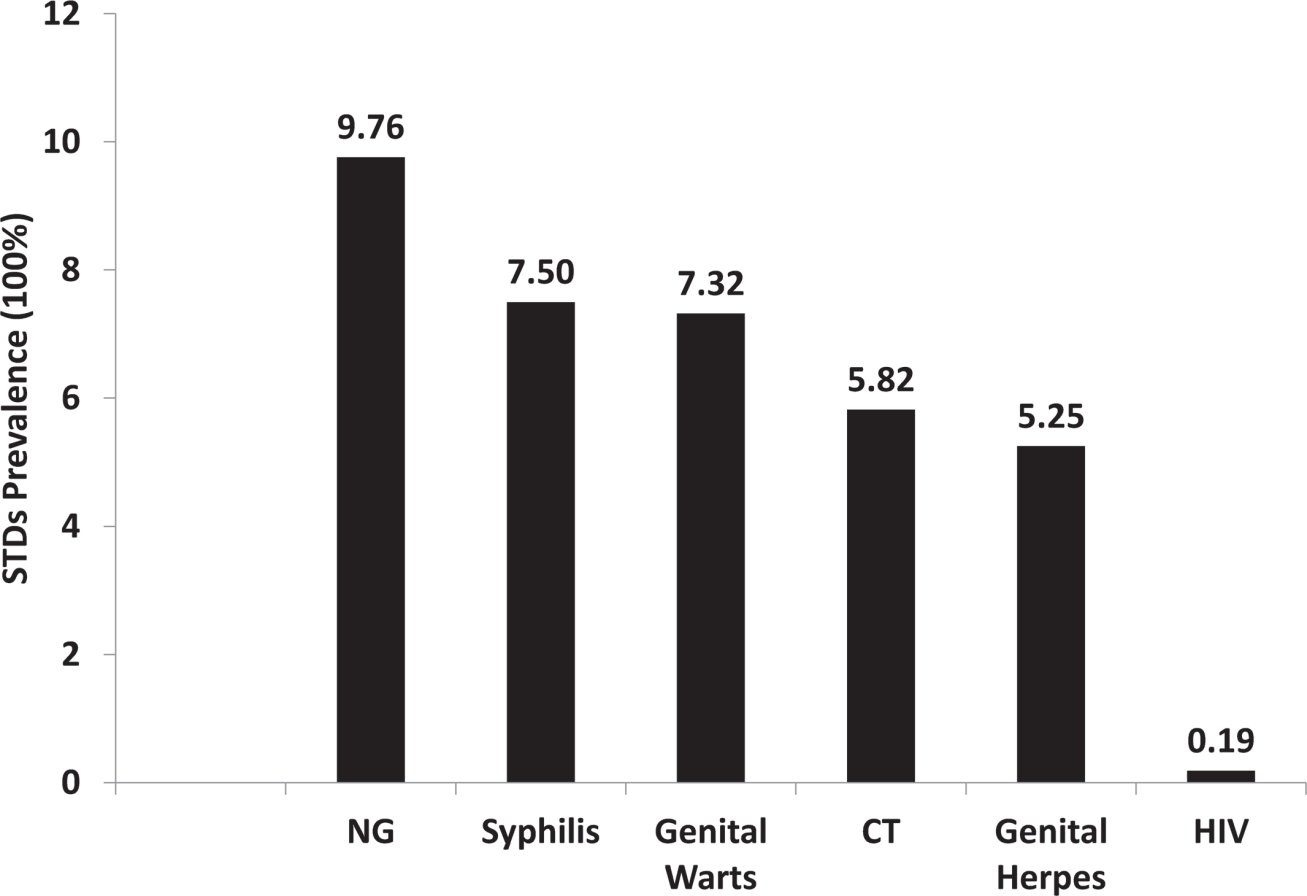
Prevalence of STDs among heterosexual males attending sexually transmitted infection clinics in Jiangmen, China, 2009 (N = 533).

In our study, 40 participants tested positive for syphilis, with syphilis prevalence of 7.50%. In addition, 39 (7.32%) and 28 (5.25%) participants were diagnosed with genital warts and genital herpes, respectively. The prevalences of genital warts among participants who living alone, living with female partners, living with others and living with family members were 5.07% (95% CI 1.36–8.78), 5.78% (95% CI 3.24–8.31), 9.52% (95% CI 0.00–23.22) and 24.44 (95% CI 11.39–37.51), respectively. Only one participant tested positive for HIV (0.19%). Among the 226 participants who tested for CT and NG, 22 (9.73%) and 14 (6.19%) were found to be positive for CT and NG (Table 2). STDs prevalence can be found in Fig. 1.

**Table 2:** Prevalence of STDs among heterosexual males attending sexually transmitted infection clinics in Jiangmen, China, 2009 (N = 533).

**Disease**		**Frequency**	**Percent (%)**
**Syphilis**	*No*	493	92.50
** **	*Yes*	40	7.50
**CT** *	*No*	204	90.27
** **	*Yes*	22	9.73
**NG** *	*No*	212	93.81
** **	*Yes*	14	6.19
**Genital Warts**	*No*	494	92.68
** **	*Yes*	39	7.32
**Genital Herpes**	*No*	505	94.75
	*Yes*	28	5.25
**HIV**	*No*	532	99.81
	*Yes*	1	0.19

Note: *226 participants were tested for CT and NG

### Factors correlated with genital warts

The results of univarate analysis indicate that age was negatively associated with risk of genital warts (cOR 0.95, 95% CI 0.91–0.99). Participants who had college degree or above and were never married had significantly higher risk of genital warts, with cORs of 1.95 (95% CI 1.00–3.90) and 3.36 (95% CI 1.73–6.54), respectively. In addition, participants who lived with family members also had higher risk of genital warts, compared with those who were lived alone. Participants who experienced STD related symptoms in the last year were also had significantly higher risk of genital warts, with cOR of 1.99 (95% CI 1.01–3.93). Similar result was also found for the participants who did not received any STD-related service in the past year (cOR 2.24, 95% CI 1.09–4.59).

The results of univariate analysis demonstrated that participants who had sex with FSWs in the last three months also had significantly higher risk of genital warts, with cOR of 2.04 (95% CI 1.04–3.98). Our study further indicated unprotected last intercourse with regular partners was positively associated with genital warts (cOR 2.20, 95% CI 1.09–4.43).

Similar results were found in the multiple logistic regressions, after adjustment for age, residence, education level and marital status. Compared to the reference groups, living with family members (versus living alone), no STD-related service in past year, experiencing STDs related symptoms in past year, and sex with FSWs in last three months were positively associated with genital warts, with adjusted ORs of 5.54 (95% CI 1.94–15.81), 2.26 (95% CI 1.08–4.74), 1.99 (95% CI 1.00–3.99) and 2.01 (95% CI 1.00–4.04), respectively. These results can be found in Table 3.

**Table 3:** Factors correlated with Genital warts among heterosexual males attending sexually transmitted infection clinics in Jiangmen, China, 2009 (N = 533).

** **	**Crude Model**				**Adjusted Model** *			
	cOR	95% CLs		P-value	aOR	95% CLs		P-value
		Lower	Upper			Lower	Upper	
**Age**	0.95	0.91	0.99	0.01				
**Education**								
*High school or below*	*Ref*							
*College or above*	1.95	1	3.8	0.05				
**Residence**								
*Migrants*	*Ref*							
*Local residence*	1.24	0.62	2.48	0.54				
**Marital status**								
*Ever Married*	*Ref*							
*Never married*	3.36	1.73	6.54	<0.001				
**Living status**								
*Living alone*	*Ref*				*Ref*			
*Live with female partners*	1.15	0.47	2.79	0.07	2.34	0.79	6.87	0.92
*Live with others*	1.97	0.38	10.19	0.97	2.67	0.49	14.53	0.87
*Live with family members*	6.06	2.18	16.79	<0.001	5.54	1.94	15.81	0.02
**Experienced listed STDs related symptoms in the last year**								
*No*	*Ref*				*Ref*			
*Yes*	1.99	1.01	3.93	0.05	1.99	1	3.99	0.05
**Received STDs related service in the past year**								
*Yes*	*Ref*				*Ref*			
*No*	2.24	1.09	4.59	0.03	2.26	1.08	4.74	0.03
**Used condom with regular female partners during the last intercourse**								
*Yes*	*Ref*				*Ref*			
*No*	2.2	1.09	4.43	0.03	1.19	0.53	2.68	0.67
**Used condom with regular female sex workers during the last intercourse**								
*Yes*	*Ref*				*Ref*			
*No*	2.05	0.84	5	0.12	1.74	0.7	4.31	0.23
**Used condom with casual female partners during the last intercourse**								
*Yes*	*Ref*				*Ref*			
*No*	1.34	0.58	3.12	0.5	1.32	0.56	3.13	0.53
**Had sex with female sex workers in the last 3 months**								
*No*	*Ref*				*Ref*			
*Yes*	2.04	1.04	3.98	0.04	2.01	1	4.04	0.05
**Had sex with casual female partners in the last 3 months**								
*No*	*Ref*				*Ref*			
*Yes*	1.82	0.89	3.74	0.1	1.92	0.92	4.02	0.08

Note:

* Model was adjusted for age (continuous), marital status, residence and education

## Discussion

Globally, much research has been directed at identifying the burden of STD infections, HPV [21,22]. However, few studies ever explored the epidemic of genital warts[15,23], and only a small number of studies have studied epidemiology of genital warts in China[12,24]. In addition, almost none have identified associated risk factors. Our study fills this gap by providing information on HIV and STDs prevalence among MSCs in China, including prevalence of genital warts. In addition, our study further explored the correlates of genital warts.

In our study, we found only one HIV positive case, and our observed HIV prevalence was lower than another study conducted among MSCs by our team in Jiangsu[10]. The syphilis prevalence of our study was much lower as well [10]. Our study also found the prevalence of genital herpes, CT and NG among MSCs in Jiangmen was high. Given the high proportions of STDs and a large number of participants engaged in unprotected sex with casual partners or FSWs, there was a potential trend for spread the epidemic of HIV among MSCs.

Our study indicates that participants who had sex with either female sex workers or casual partners had higher risk of genital warts. These findings agree with previous studies that demonstrated that men who had sex with FSWs or casual partners had higher risk of HPV infection[25,26], and risk increases with each subsequent partner[22]. As a large proportion of MSCs engaged in both commercial sex and casual sex in the last three months, increased attention should be paid to them.

Our study did not find significant correlation between last unprotected vaginal sex (UVI) in the last three months and the risk of developing genital warts. There were several potential reasons for this phenomenon. First, the median incubation period for genital warts is around three months (ranged from one to eight months)[27], and our studied only assessed sexual behavior within the last three months. Even if participants were infected with HPV during their last unprotected intercourse they would still be asymptomatic at the time of the study. The recurrence of genital warts could be the second reason for these non-significant correlations, as the relapse rate is high after the initial treatment [28]. Third, even thought there may have strong correlation between unsafe sex and HPV infection among MSCs [21], the correlation between unprotected sex and genital warts may be weak, as only a small portion of people develop genital warts after HPV infection.

After adjusted for age and marital status, living with family members was still found to positively correlate with genital warts. The reason behind this phenomenon is still unknown. One potential explanation is these participants tend to have high risky behaviors like have sex with FSWs or casual partners in our study.

Our study indicated that a large proportion of MSCs in Jiangmen engaged in UVI during last vaginal intercourse. This proportion was even higher for UVI with casual partners. Our findings were different from previous studies, which reported that MSCs have higher UVI rate with regular partners[29]. One potential reason for the phenomenon is we only measured UVI during last intercourse, instead of last three months or six months.

Our study has several limitations. First, this study was conducted at STD clinics of public hospitals, which may lead to potential selection bias. Some patients who were worried about the disclosure of their STDs status may not come to the public STD clinics, and this group of population may differ from the participants of our study. Second, behaviors were measured by self-report, which may lead to a social desirability bias. Third, as a cross-sectional study, our study has the problem of time ambiguity, since the outcome and potential exposures were almost measured at the same time. Thus, all of the findings identified in our studies need to explained by causation, and no casual inference could be drawn based on our studies.

### Conclusion

Our study indicates that the prevalence of STDs among MSCs in Jiangmen was high, which may further spread HIV among MSCs. In addition, the development of genital warts was positively correlated with lack of services, experienced STDs related symptoms in the last year, living with family members and engaging in commercial sex in the last three months. Targeted interventions that focused on STDs related services uptake (include but not limit to condom distribution and promotion, more frequent STDs testing and standard treatment) should be implemented urgently.

## Supporting Information

S1 Dataset Original data set. (XLS)
